# Higher Medical Education

**Published:** 1908-07

**Authors:** E. R. Anthony

**Affiliations:** Griffin, Ga.


### HIGHER MEDICAL EDUCATION.

BY E. R. ANTHONY, M. D., GRIFFIN, GA.

During the last two decades medicine has made wonderful progress. The benefits conferred on the human race by her discoveries in that time have been enormous and have added greatly to the health and wealth of communities and to the happiness and safety to individuals. The great State of Georgia should and will see the importance of the accomplishments of modern medicine and should give us every encouragement and assistance in the struggle for still greater things. We do not ask for money, but we do ask for fair and just legislation that will enable us to reach higher standards of medical education and thereby enable us to solve many important problems now in sight With all due deference to the grand men and noble characters who worked and toiled in our profession twenty-five or thirty years ago—for I yield to none in my admiration for their devotion to our profession and their skill in its practice—I must say that modern medicine requires a better order of intellect, a higher preliminary education, and a more thorough professional training than it did twenty-five years ago. The weakest part of our system of medical education today is the low standard of preliminary requirements. This standard can not be raised except by legislation. The desire for higher standards, both for preliminary education and medical training has been manifest in all sections of the country. The public holds the medical profession to a strict account for any abuses which occur in connection with medical affairs, and the laity looks to the medical profession to bring about better standards of medical education. The public is already demanding such laws that will assure the adoption and enforcement of higher standards and the legislators who enact our laws are ever ready to listen to public demands. For whom is medical legislation desired? Is it for the benefit of the medical profession and its individual members or is it for the benefit of the public? If it is for the benefit of the profession only, it falls under the head of *class legislation* and no member of the profession would have the gall to work for it, and no legislator would be brave enough to face the frowns of his constituents and aid us in our requests and demands for it. Every thinking man must now admit that all medical legislation must find its ultimate reason in the benefit to the public and the good to the community at large which it produces. This statement is so plain, so patent that it needs no argument for its proof. Under the Federal Constitution the regulation of the medical profession and its relation to the public comes under the police power of the state and is not a function of the general government. There are at present fifty-two political divisions in the United States. The legislative body of each of these divisions has a right to adopt such legislaiton and enact such laws as it may see fit, to regulate the practice of medicine in her borders. All of these political divisions (48 states and 4 territories) with the exception of Alaska, have a medical law and licensing board. In several of the states there in four distinct lines of statutory requirements; (1) Preliminary Education, (2) Medical Education, (3) Licensing Test, (4) Registry of License. A large majority of the states have the last three requirements and all have the last two. Quite a number of the states have the first requirement and in a majority of the states legislation requiring a preliminary education is now pending. The present medical law of Georgia was passed in 1894, nearly fifteen years ago. In that time many changes have taken place and medicine has made a rapid progress, thereby necessitating many changes in her law. In analysing the medical practice act of Georgia, we find that the most salient feature will come under the following heads; (1) Character and Composition of Medical Licensing Board, (2) The manner of Appointing Licensing Board, (3) Definition of the Practice of Medicine, (4) Requirements for application for License, (5) County Registration of License, (6) Exemptions, (7) Penalty, (8) Reciprocity. In my opinion, we should ask for no changes in this law under heading *one*. While I think that it would be desirable to have but one licensing board in Georgia with a fair and equitable representative from each school, (Regular, Eclectic and Homeopathic). I do not think it expedient at this time to ask for any change in that direction. Under heading *two*, I think there should be a change. The appointment of the members of the Board should be kept out of politics. The appointments should be made by the Governor from a list of names selected by the Medical Association of Georgia. Under heading *three* there is no urgent necessity for a change. While the law is not as comprehensive as it should be in regard to the definition of the practice of medicine, we can afford to let it remain as it now stands. Under heading *four* there is a crying need of a radical change. Georgia is the only state in the Union that allows a graduate of a school requiring only three year courses of lectures to apply for examination and license. As a matter of course this should be changed to four years. But the change, above all others, that should be made under this heading is a *preliminary education requirement.* The applicant for license should have documentary evidence of at least a high school education, or he should be examined in such branches as are taught in the high schools, by the Board. This would, sooner or later, force the Medical Coellges to matriculate no scholar having less than a high school education. Headings five, six, seven and eight are about as near perfect as we can get them. I now beg to suggest several amendments to our law. While the changes above suggested and the amendments to be suggested would by no means make our law an ideal one, they would make a great improvement upon the present one law. In the manner of obtaining a temporary license we need a change. Instead of requiring a young doctor to travel all over the state at considerable expense, loss of time and inconvenience, to be examined for temporary license, allow him to be examined either by the President or the Secretary of the Board for license. Another change in the interest of the applicant for license would be the division of the licensing examination into two parts, permitting the applicant to be examined at the end of the Sophomore year upon the subjects he had been taught in the first two years. In Great Britain there are four separate examinations and in France there are five. Another desirable change is this: The Board should be given the power to revoke a license for just cause, such as habitual drunkenness, immoral conduct, producing abortion, fradulently obtaining a license and the like. Another amendment should be passed authorizing the Board together with the President of the Board of Counsellors of the Medical Association of Georgia to determine *good standing* of Medical Colleges. There are now in the United States about one hundred and sixty Medical Colleges, more than all the countries of Europe combined have. A goodly number of these are nothing more than diploma mills and should not be recognized by the Board.

In conclusion, permit me to say, that if we do not keep the appointment of the Board out of the mire of politics and the Medical Colleges out of the slums of commercialism the standard of medical education will never be elevated to a much higher plane than it now occupies.

